# Preventive and Therapeutic Effects of Crocetin in Rats with Heart Failure

**DOI:** 10.3390/ph17040496

**Published:** 2024-04-12

**Authors:** Renqiang Ma, Sijia Li, Qingmei Mo, Xiaojuan Chen, Yan Liang, Tao Hu, Hui Hu, Bao He, Renshi Li, Junping Kou, Boyang Yu

**Affiliations:** 1 Jiangsu Key Laboratory of TCM Evaluation and Translational Research, Research Center for Traceability and Standardization of TCMs, School of Traditional Chinese Pharmacy, China Pharmaceutical University, 639 Longmian Road, Nanjing 211198, China; pony213@163.com (R.M.); 19850858730@163.com (Q.M.); 15907650622@163.com (X.C.); junpingkou@cpu.edu.cn (J.K.); 2 Boji Pharmaceutical Research Center, Boji Medical Biotechnological Co., Ltd., Guangzhou 510663, China; 15196643789@163.com (S.L.); shijianyu@126.com (Y.L.); gdpuht0918@163.com (T.H.); huhui1219@126.com (H.H.); hb_nj@163.com (B.H.)

**Keywords:** chronic heart failure, crocetin, load-induced heart failure, myocardial ischemia

## Abstract

Gardenia is both a food and medicine plant. It is widely used for cardiovascular protection, and its main bioactive ingredient is crocetin. This study aims to observe the therapeutic effects of crocetin on chronic heart failure in rats induced by various etiologies. It further compares the efficacy differences between preventative and treatment administration, varying dosages, and treatment durations, to provide improved guidance for medication in heart failure rats and determine which categories of chronic heart failure rats might benefit most from crocetin. Chronic heart failure models induced by abdominal aorta constriction, renal hypertension, and coronary artery ligation were constructed. By examining cardiac function, blood biochemistry, and histopathology, the study assessed the preventive and therapeutic effects of crocetin on load-induced and myocardial ischemia-induced heart failure. The results showed that in all three models, both treatment and preventative administration of crocetin significantly improved chronic heart failure in rats, especially in preventative administration. The results indicate crocetin may be beneficial for improving symptoms and functional capacity in rats with heart failure. Furthermore, long-term administration was more effective than short-term administration across all three rat models, with therapeutic onset observed over 6 weeks.

## 1. Introduction

Gardenias, belonging to the Rubiaceae family, are derived from the dried, mature fruits of *gardenia jasminoides* Ellis. The blossoms of gardenia are either white or yellow, exuding a rich fragrance, while the fruit is typically yellow or orange-red, and either oval or elongated in shape [1]. There are approximately over 200 varieties of gardenias worldwide, predominantly found in the regions of Jiangxi, Hunan, and Fujian in China [2]. Due to their wide distribution, they are readily accessible. Gardenia is recognized as both a food and a medicinal herb in traditional Chinese medicine, containing various chemical compounds with wide applications such as natural food colorants, traditional Chinese pharmaceuticals, and health supplements [2]. Gardenia yellow pigment is a water-soluble natural dye primarily extracted from the fruit, celebrated for its safety and certain nutritional and health benefits. It is particularly noted for its effective coloring properties on proteins and starches, thereby extensively used in a myriad of food products including cakes, candies, flour, beverages, and jellies [3,4]. The primary components of gardenia yellow pigment are crocins and crocetin. Crocetin (CRA) is reported to exhibit a wide range of pharmacological activities including anti-tumor [5,6,7,8], cardioprotective [9,10], anti-thrombotic [5], anti-ischemic brain injury [11,12], antihyperglycemic [13], antilipemic [13], anti-atherosclerotic [14], antioxidant [15,16,17], and anti-free radical properties [18]. The extensive potential of CRA indicates its promising prospects for further research and application.

Heart failure (HF), also known as cardiac insufficiency, is a syndrome where the heart’s pumping capacity is compromised due to impairments in its contractile and/or relaxative functions. This leads to inadequate expulsion of venous blood returning to the heart, resulting in reduced cardiac output, venous congestion, and insufficient arterial blood perfusion [19,20]. These dysfunctions culminate in a syndrome of circulatory disturbance characterized clinically by congestion in the pulmonary and/or systemic circulation and insufficient tissue blood perfusion. According to the “AHA/ACC/HFSA Heart Failure Diagnosis and Treatment Guidelines”, heart failure is categorized based on the left ventricular ejection fraction (LVEF) into heart failure with reduced ejection fraction (HFrEF), Heart Failure with preserved ejection fraction (HFpEF), and heart failure with mid-range ejection fraction (HFmrEF) [21]. Each type reflects a different aspect of the heart’s inability to function effectively, necessitating targeted approaches to management and treatment.

The pathogenesis of heart failure is complex and involves many aspects, including lipid metabolism disorders, mitochondrial damage, oxidative stress, and other factors. It has been reported that in the early and middle stages of heart failure, myocardial energy metabolism can be maintained relatively normal, the proportion of *β*-oxidation of the main fuel fatty acids is unchanged or slightly increased, and glucose uptake rate and glycolysis are also increased to maintain energy supply [22]. With the development of heart failure, the dynamic balance of fatty acid oxidation, aerobic oxidation of glucose and glycolysis is gradually destroyed. In the end stage of heart failure, the utilization of fatty acids by cardiomyocytes decreases, leading to an increase in free fatty acids in the blood. The accumulation of ceramide, diacylglycerol, and other lipotoxic substances in cardiomyocytes will cause oxidative stress damage, destroy the structure and function of mitochondria, and promote apoptosis [23]. In addition, during the development of heart failure, reduced fatty acid oxidation leads to the accumulation of lipotoxic metabolites and the production of a large number of reactive oxygen species. ROS can cause calcium overload in cardiomyocytes and promote cytochrome c-catalyzed oxidation of cardiolipin, which may impair mitochondrial fatty acid oxidation. This vicious cycle will further stimulate cardiomyocyte apoptosis and aggravate the course of heart failure [24]. Therefore, improving heart failure by regulating energy metabolism is one of the important treatment directions.

With the development in the pathogenesis of heart failure, significant progress has been made in pharmacological treatments, ushering in an era of multi-mechanism and multi-target strategies. These include Angiotensin Receptor Neprilysin Inhibitors (ARNI), Sodium-Glucose Co-Transporter 2 Inhibitors (SGLT-2), soluble Guanylate Cyclase Stimulators such as Vericiguat, If channel inhibitors like Ivabradine, selective cardiac myosin activators [25,26] (Omecamtiv Mecarbil, OM), etc. The therapeutic agent’s choice varies with the stage of heart failure. For instance, HFrEF management predominantly involves ARNI, SGLT-2, OM, and Vericiguat, while HFpEF stages often use SGLT-2 inhibitors like Empagliflozin and Dapagliflozin [27]. Beyond these pharmaceuticals, traditional Chinese medicine also plays a significant role in heart failure treatment, known for its synergistic actions at multiple sites. Traditional Chinese medicine typically exerts effects through antioxidative stress, anti-inflammatory actions, energy metabolism regulation, ventricular remodeling improvement, myocardial fibrosis reduction, and angiogenesis promotion [28,29,30]. Consequently, numerous traditional Chinese medicine preparations have emerged as treatments for heart failure, including Shengmai Drink, Yixinshu Capsules, Qili Qiangxin Capsules, and Nuanxin Capsule [31,32,33]. These illustrate the growing diversification and sophistication of heart failure treatment modalities.

Crocetin exhibits multiple pharmacological effects, as follows. Anti-inflammatory effects: CRA exerts anti-inflammatory effects by inhibiting the adhesion and infiltration of immune cells to the endothelium in the lipopolysaccharide-induced human umbilical vein cell inflammation model [34]. Regulation of tissue oxygen content: CRA promotes brain tissue oxygenation under vascular occlusion conditions while attenuating brain tissue hyperoxygenation during vascular perfusion [35]. Cardioprotective effect: CRA can alleviate oxidative stress, apoptosis, and mitochondrial dysfunction in myocardial tissue of acute myocardial ischemia and the heart injury reperfusion model via the SIRT3/FOXO3a/SOD2 signaling pathway [36]. In the aortic band myocardial hypertrophy model, CRA can improve myocardial hypertrophy, fibrosis and inflammation [10]. CRA can also improve myocardial remodeling by increasing ATPase activity, decreasing collagen content and inhibiting MMPs activity in the abdominal aortic coarctation-induced rat cardiac hypertrophy model [37]. On the basis of previous reports, CRA was found to improve cardiac hypertrophy and protect against myocardial ischemia and reperfusion injury. Therefore, it is speculated that CRA has a certain beneficial effect on heart failure, and there is less research content on heart failure caused by different etiology. On this basis, the present study systematically explored the preventive and therapeutic effects of CRA on chronic heart failure models caused by load heart failure and coronary artery ligation, observed the preventive and therapeutic effects of CRA on chronic heart failure caused by different causes, and explored the clinical administration regimen so as to provide an experimental basis for the clinical application of CRA in the prevention and treatment of chronic heart failure.

## 2. Results

### 2.1. Preventive and Therapeutic Effects of CRA on Chronic Heart Failure Caused by Abdominal Aortic Coarctation

#### 2.1.1. CRA Can Improve Cardiac Function in Rats with Chronic Heart Failure Caused by Abdominal Aortic Coarctation

**Figure 1:**
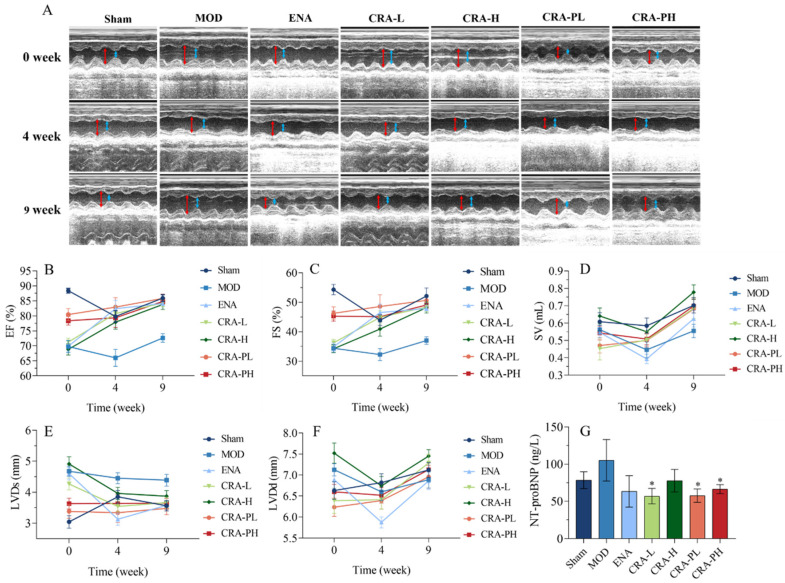
CRA can improve cardiac function in chronic heart failure caused by abdominal aortic coarctation. The cardiac function parameters were measured at 0, 4, and 9 weeks of administration for the CRA treatment group. However, for the CRA prevention group, they were measured at 8, 12, and 17 weeks (**A**–**F**). (**A**) In the image of the ultrasonic cardiogram, the red arrowhead shows LVDd, blue arrowhead shows LVDs. (**B**) Ejection fraction, (**C**) short-axis shrinkage, (**D**) stroke output, (**E**) left ventricular end-systolic diameter, (**F**) left ventricular end-diastolic dimension, (**G**) NT-proBNP was detected at the end of administration. * *p* < 0.05 vs. MOD. *n* = 10 per group.

In the abdominal aorta-induced heart failure model, there were significant decreases in both ejection fraction (EF) and fractional shortening (FS) post-modeling (Figure 1B,C), and a significant enlargement in left ventricular systolic dimension (LVDd) and left ventricular diastolic dimension (LVDs) (*p* < 0.01) (Figure 1A,E,F). After 4 weeks of CRA treatment, the CRA-L group showed a significant increase in EF (*p* < 0.05). After 9 weeks of treatment, both EF and FS significantly increased (*p* < 0.01). Meanwhile, LVDs and LVDd in all CRA treatment groups (low-dose *p* < 0.05, high-dose *p* < 0.01) significantly decreased. With preventative administration of CRA for 8, 12, and 17 weeks, both EF and FS significantly increased at all time points (*p* < 0.01). Also, the preventative treatment group showed a significant reduction in LVDs and LVDd at 8 and 17 weeks (*p* < 0.01). Thus, both preventative and therapeutic dosing of CRA markedly improved cardiac ejection fraction and contractile function, with preventative treatment showing more pronounced effects, suggesting its potential for better outcomes in chronic heart failure management.

Stroke volume (SV) is a critical indicator of cardiac pump function, related to factors such as myocardial contractility, venous return, and arterial pressure. Generally, SV increases with enhanced myocardial contractility and venous return but decreases with higher arterial pressure. After modeling, SV decreased, but following 9 weeks of treatment with high-dose CRA, there was a significant increase in SV (*p* < 0.01), with other treatment groups showing an increasing trend (Figure 1D).

Brain Natriuretic Peptide (BNP) and N-terminal pro-brain natriuretic peptide (NT-proBNP) are members of the natriuretic peptide family [38,39] which are released in response to myocardial stretch or increased ventricular wall stress. NT-proBNP is considered a more sensitive marker for early cardiac dysfunction compared to BNP due to its higher stability [40,41], lesser individual variability, and higher concentration in blood, making it a preferred marker. It was shown that while the model control (MOD) group exhibited an upward trend in NT-proBNP levels (Figure 1G), all other groups demonstrated a downward trend, indicating that CRA could ameliorate heart failure induced by the abdominal aorta to some extent.

#### 2.1.2. CRA Can Improve Myocardial Hypertrophy in Chronic Heart Failure Rats Caused by Abdominal Aortic Stenosis

**Figure 2:**
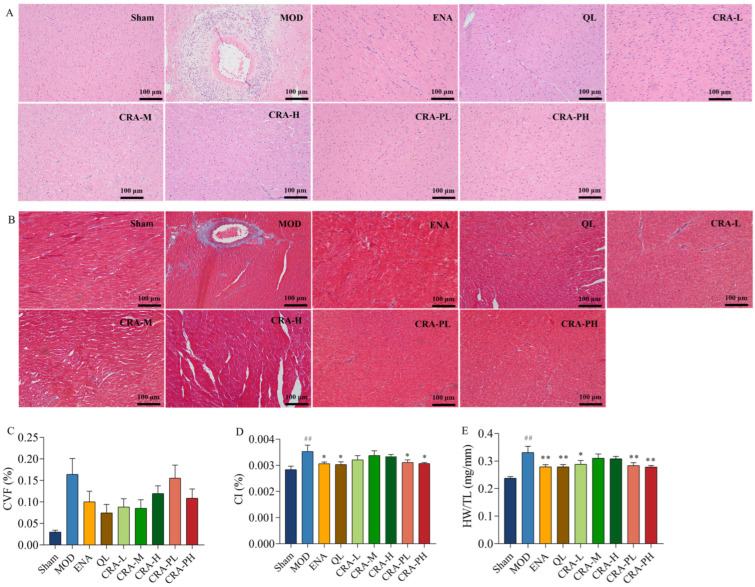
CRA can alleviate organic damage in chronic heart failure caused by abdominal aortic coarctation. The left ventricle sections were conducted with H&E staining and Masson’s trichrome staining (**A**,**B**), Scale bars, 100 μm. (**A**) H&E staining, (**B**) Masson’s trichrome staining, and (**C**) Collagen volume fraction were calculated by three images of the infarct area, normal and infarct borderline area, and normal area. Myocardial hypertrophy was indicated in (**D**). (**D**) CI was calculated by the ratio of heart weight to body weight, and (**E**) Heart weight to tibial length ratio. ## *p* < 0.01 vs. Sham; * *p* < 0.05, ** *p* < 0.01 vs. MOD. *n* = 10 per group.

The analysis of the Cardiac Index (CI) and Heart Weight to Tibia Length ratio (HW/TL) revealed that both metrics were significantly elevated in the model control group (*p* < 0.01), indicating myocardial hypertrophy post-abdominal aorta modeling. After 9 weeks of CRA treatment, the CRA-L group (Figure 2D,E) showed a significant reduction in HW/TL (*p* < 0.05) and a downward trend in CI. The preventative CRA treatment groups demonstrated a significant decrease in both HW/TL and CI (HW/TL, *p* < 0.01; CI, *p* < 0.05). These results suggest that both CRA treatment and preventative groups can mitigate myocardial hypertrophy, with the preventative group showing superior efficacy.

#### 2.1.3. CRA Can Reduce Myocardial Fibrosis in Abdominal Aortic Coarctation-Induced Chronic Heart Failure Rats

The study also explored the effects of CRA on myocardial fibrosis in chronic heart failure induced by abdominal aorta constriction. H&E and Masson staining results showed that the sham-operated group had clear and orderly myocardial fiber structures. In contrast, the model control group exhibited severe pathology with extensive inflammatory infiltration, myocardial cell necrosis, proliferating fibrous tissue, and instances of myocardial cell hypertrophy. Post CRA administration, there was a notable improvement in inflammatory infiltration across all groups, with no apparent myocardial cell necrosis and a decrease in fibrosis levels. Statistical analysis of the myocardial collagen volume fraction indicated a decreasing trend in all CRA and ENA groups. These results revealed that CRA can alleviate myocardial fibrosis, reducing inflammation and myocardial hypertrophy.

### 2.2. Preventive and Therapeutic Effects of CRAT on Heart Failure Rats Caused by Renal Hypertension

#### 2.2.1. CRAT Can Reduce Blood Pressure

**Figure 3:**
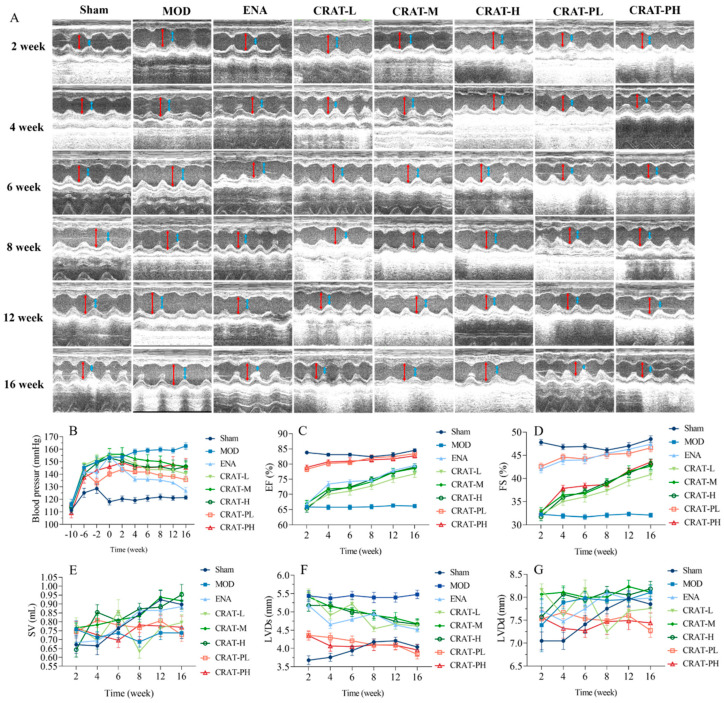
CRAT can lower blood pressure and improve cardiac function of chronic heart failure due to renal hypertension. The cardiac function parameters were measured at 2, 4, 6, 8, 12, and 16 weeks of administration for the CRAT treatment group. The CRAT prevention group was measured at 12, 14, 16, 18, 22, and 26 weeks (**A**,**C**–**G**). (**A**) In the image of ultrasonic cardiogram, the red arrowhead shows LVDd, blue arrowhead shows LVDs. (**B**) Blood pressure was measured at 10, 6, and 2 weeks before modeling and 0, 2, 4, 6, 8, 12, and 16 weeks of administration for the CRAT treatment group. However, for the prevention group, it was measured from 0 weeks to 26 weeks. The cardiac function parameters were measured at 2, 4, 6, 8, 12, and 16 weeks (**B**–**G**). (**C**) Ejection fraction, (**D**) Short-axis shrinkage, (**E**) Stroke output, (**F**) Left ventricular end-systolic dimension, (**G**) Left ventricular end-diastolic dimension. *n* = 10 per group.

After four weeks post-modeling, all groups except the sham-operated group exhibited a significant increase in blood pressure (Figure 3B, *p* < 0.01). After 4 weeks of treatment, there was no significant change in blood pressure; however, after 8 weeks of treatment, the CRAT-PL showed a significant reduction in blood pressure (*p* < 0.01). After 10 weeks of modeling, both CRAT-PL and CRAT-PH showed a significant reduction in blood pressure (*p* < 0.01).

These results indicated that administering CRAT leads to a blood pressure decrease in both treatment and preventative groups, with more extended treatment periods resulting in more significant reductions. Thus, CRAT can suppress the blood pressure increase caused by the two-kidney one-clip model, and providing CRAT early can suppress the rise in blood pressure, potentially delaying the progression of heart failure development.

#### 2.2.2. CRAT Can Improve Cardiac Function in Heart Failure Rats Caused by Renal Hypertension

The study results indicated that post-modeling, cardiac contractile function weakened, as evidenced by a significant decrease in ejection fraction (EF) and fractional shortening (FS) (*p* < 0.01). After 2 weeks of treatment, the preventative treatment group exhibited a significant decrease in LVDs and a significant increase in EF and FS (Figure 3C,D,F,G). After 16 weeks of CRAT administration, the treatment group showed a significant decrease in LVDs (*p* < 0.01), and the preventative group exhibited significant reductions in both LVDs and LVDd (*p* < 0.01), along with a significant increase in EF and FS (*p* < 0.01). These results suggest that CRAT can improve cardiac contractile function and increase the ejection fraction, with effects becoming more pronounced over time. Moreover, preventative CRAT administration can improve ejection function faster and more effectively than treatment administration.

**Figure 4:**
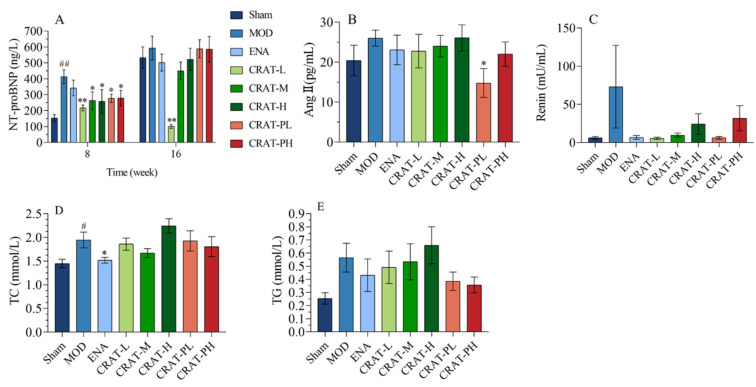
CRAT may relieve heart failure by inhibiting the renin-angiotensin system and regulating lipid metabolism. (**A**) NT-proBNP was measured at 8 and 16 weeks of administration for the CRAT treatment group. But for the prevention group, it was measured at 18 and 26 weeks. (**B**,**C**) were designed to observe whether CRAT can improve heart failure through the renin-angiotensin system. (**B**) Serum of AngII and (**C**) Renin were measured. (**D**)Serum of TCH, (**E**) TG were measured. # *p* < 0.05, ## *p* < 0.01 vs. Sham; * *p* < 0.05, ** *p* < 0.01 vs. MOD. *n* = 6 per group.

In the renal hypertension model, increased blood pressure leads to greater peripheral resistance, and the modeling causes a decrease in myocardial contractility, leading to reduced SV (Figure 4E). From 2 to 16 weeks post-treatment, the model group’s SV maintained at about 0.7–0.8 mL. After CRAT administration, all groups showed an increasing trend in SV, with the CRAT-H and CRAT-M groups’ SV nearly aligning with the sham-operated group at 16 weeks. This indicates that CRAT can effectively mitigate the reduction in SV caused by increased blood pressure and weakened myocardial contractility in the renal hypertension model.

#### 2.2.3. CRAT May Reduce the Degree of Heart Failure by Inhibiting the Renin-Angiotensin System and Improving Lipid Metabolism

After 18 weeks of modeling, compared to the sham-operated group, the model control group (Figure 4A) showed a significant increase in NT-proBNP. Compared to the model control group, the treatment and preventative treatment groups all showed a significant decrease in NT-proBNP after 8 weeks of treatment and there was a significant reduction in the CRAT-L group after 16 weeks of treatment.

The renin-angiotensin system (RAS) plays an important role not only in traditional hemodynamic functions such as sodium retention and increasing vascular resistance but also in paracrine and autocrine functions in the heart [42]. These latter functions are critical in ventricular remodeling and the development of congestive heart failure. Angiotensin II (AngII), a potent vasoconstrictor and an active component of RAS, has significant implications in the progression of chronic heart failure, including vasoconstriction and increasing cardiac afterload. Also, AngII would promote the release of norepinephrine from sympathetic nerve terminals leading to elevated catecholamine levels and stimulate aldosterone release leading to increased blood volume and cardiac preload. Meanwhile, AngII can induce myocardial and interstitial cell proliferation and hypertrophy and lead to left ventricular remodeling [43,44]. In this study, the model control group showed an increasing trend in AngII and renin levels, while the CRAT-PL group showed a significant decrease in AngII, with other treatment groups also exhibiting decreasing trends. All groups showed a decreasing trend in renin levels (Figure 4B,C), suggesting that CRAT preventative treatment might inhibit the production of AngII, thereby alleviating heart failure.

The results also showed that the model control group (Figure 4D,E) had a significant increase in total cholesterol (TC) (*p* < 0.05) and an upward trend in triglycerides (TG). After CRAT administration, except for the high-dose group, all other treatment groups showed a decreasing trend in TC, with the positive control group showing a significant reduction (*p* < 0.05). This suggested that CRAT can regulate lipid metabolism to some extent, with CRAT-PL and CRAT-PH being more effective in reducing serum triglyceride levels compared to the treatment group.

#### 2.2.4. CRAT Can Improve Myocardial Hypertrophy and Pathological Damage Caused by Heart Failure Caused by Renal Hypertension

**Figure 5:**
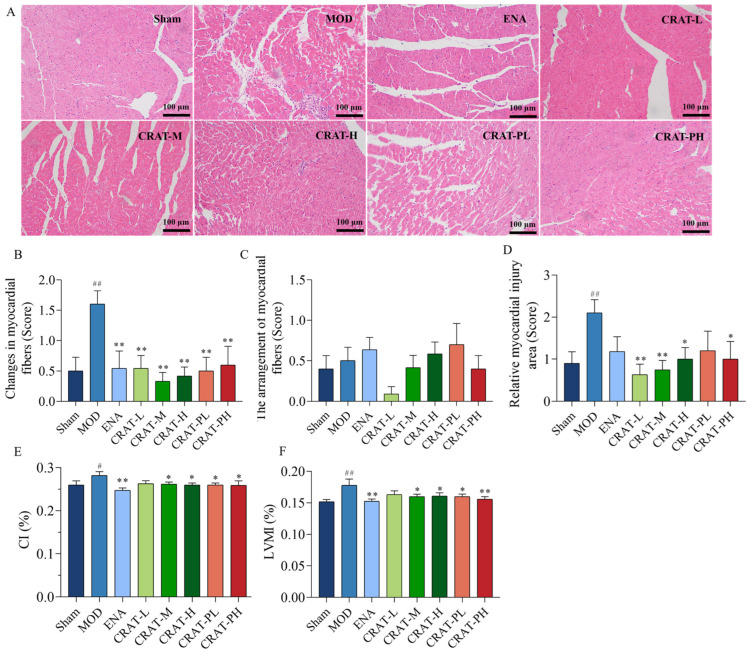
CRAT can improve the organic damage of heart failure caused by renal hypertension. The left ventricle sections underwent H&E staining (**A**), and (**B**) with corresponding quantification data of heart injury. Scale bars, 100 μm. (**C**,**D**) reflect myocardial hypertrophy, (**E**) CI was calculated by the ratio of heart weight to body weight, and (**F**) LVMI index was calculated by the ratio of left ventricle mass to body weight. # *p* < 0.05, ## *p* < 0.01 vs. Sham; * *p* < 0.05, ** *p* < 0.01 vs. MOD. *n* = 10 per group.

Post-modeling, the heart underwent remodeling, with a significant increase in the cardiac mass index and ventricular mass index. After administering CRAT, the CRAT-M, CRAT-H, CRAT-PL, and CRAT-PH groups showed a significant reduction in both Cardiac Index and ventricular mass index, indicating that CRAT can inhibit myocardial remodeling and mitigate myocardial hypertrophy (Figure 5C,D).

Myocardial fibrosis is caused by myocardial injury, where the interstitial spaces become filled with inflammatory cells, and the dead myocardial cells are replaced by collagen components secreted by fibroblasts, leading to the development of myocardial fibrosis [45]. H&E staining (Figure 5A,B) results showed that the model control group had various inflammatory cells infiltrating the myocardial interstitial spaces, with widespread necrosis and a large area of myocardial damage, including myocardial fibrosis in the infarcted regions. Compared to the model control group, all CRAT treatment groups showed reduced myocardial inflammation and a significant decrease in myocardial damage area, suggesting that CRAT can suppress inflammation, improve myocardial fibrosis, and alleviate myocardial injury.

### 2.3. Preventive and Therapeutic Effects of CRAT on Chronic Preserved Heart Failure Rats Induced by Coronary Artery Ligation

#### 2.3.1. CRAT Can Improve Cardiac Function in Heart Failure Rats with Chronic Preserved Heart Failure Caused by Coronary Artery Ligation

**Figure 6:**
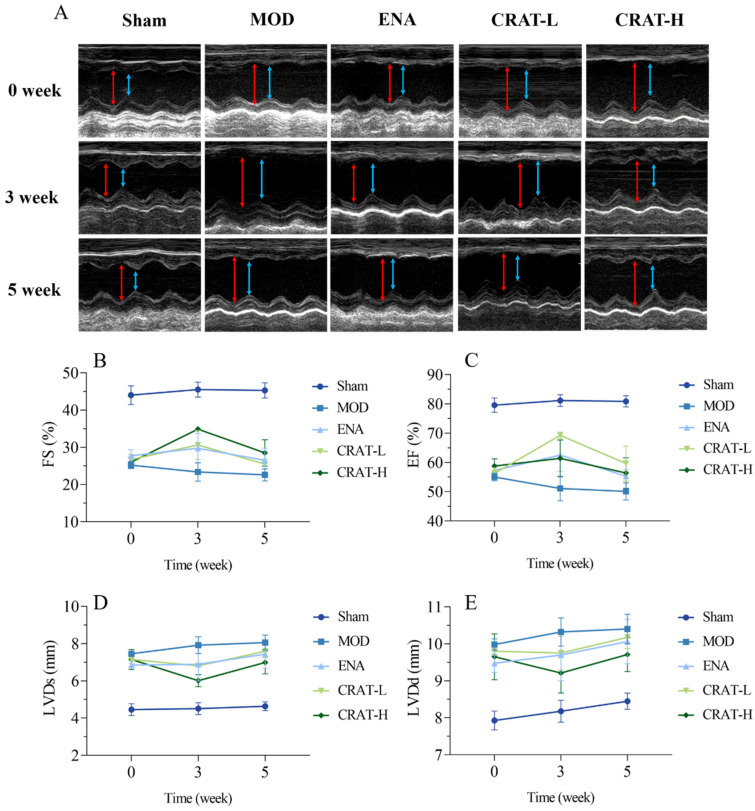
CRAT can improve cardiac function of chronic heart failure caused by coronary artery ligation. The cardiac function parameters were measured at 0, 3, and 5 weeks of administration (**A**–**E**). (**A**) In the image of the ultrasonic cardiogram, the red arrowhead shows LVDd, blue arrowhead shows LVDs. (**B**) Ejection fraction, (**C**) short-axis shrinkage, (**D**) stroke output, (**E**) left ventricular end-systolic dimension. *n* = 4 per group.

At 8 weeks post-modeling, compared to the sham-operated group, the model control group (Figure 6) exhibited a significant decrease in EF and FS (*p* < 0.01) and a significant increase in LVDd and LVDs (*p* < 0.01). This suggests that the model animals experienced an enlargement of the left ventricular cavity and impaired contractile function. After 3 weeks of treatment, the CRAT-H group showed a significant reduction in LVDs (*p* < 0.05), and both the CRAT-L and high-dose CRAT groups exhibited significant increases in EF and FS (*p* < 0.01). The results indicated that CRAT can significantly improve left ventricular cavity size and ejection function in rats with heart failure, thereby serving as an effective treatment. After 5 weeks of treatment, all treatment groups showed a downward trend in LVDd and LVDs and an upward trend in EF and FS. However, the improvement was less pronounced compared to the 3-week mark, possibly due to the progression of cardiac injury over time, and the 5-week treatment period was not sufficient to significantly ameliorate the cardiac ejection function.

#### 2.3.2. CRAT Can Improve Energy Metabolism and Lipid Metabolism

The CRAT-H group showed a decreasing trend in NT-proBNP levels after 5 weeks of treatment, suggesting that CRAT can ameliorate chronic heart failure caused by myocardial ischemia.

**Figure 7:**
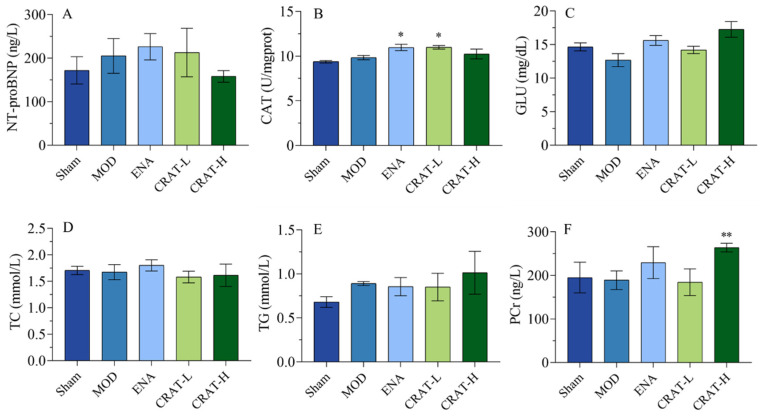
CRAT can improve energy metabolism and lipid metabolism. (**A**) The serum of NT-proBNP concentration, (**B**) the concentration of CAT in myocardial tissue, (**C**) serum GLU, (**D**) serum TCH, and (**E**) serum TG were measured. (**F**) The concentration of PCr in myocardial tissue. * *p* < 0.05, ** *p* < 0.01 vs. MOD. *n* = 8 per group.

Catalase (CAT) is a key enzyme that decomposes hydrogen peroxide into water and oxygen, serving as a major component of the cellular antioxidant defense system [46]. Oxidative stress leads to an increase in hydrogen peroxide levels. CAT helps mitigate lipid peroxidation-induced damage by scavenging hydrogen peroxide. The positive control group and CRAT-L group both showed a significant increase in CAT activity, with the CRAT-H group exhibiting an increasing trend (Figure 7B). These results suggested that CRAT can elevate CAT activity, thereby reducing myocardial oxidative stress damage.

The results (Figure 7C–E) also indicated a trend toward decreased glucose (GLU) levels in the model control group, while the ENA, CRAT-L, and CRAT-H groups all showed increasing trends. There was no significant change in TC levels in the model control group, while the CRAT-L and CRAT-H groups showed a decreasing trend. The model control group’s TG showed an increasing trend, while the CRAT-H group showed an increasing trend compared to the model control group, with no significant changes in other groups.

Phosphocreatine (PCr) is a reserve of energy in the myocardium used for ATP resynthesis, providing energy for myocardial contraction and improving symptoms of heart failure [47]. With the administration of CRAT, there was an increase in myocardial PCr content (Figure 7F), suggesting a protective effect on the myocardium and an improvement in heart failure symptoms.

#### 2.3.3. CRAT Can Improve Myocardial Hypertrophy Caused by Chronic Heart Failure Induced by Coronary Artery Ligation

**Figure 8:**
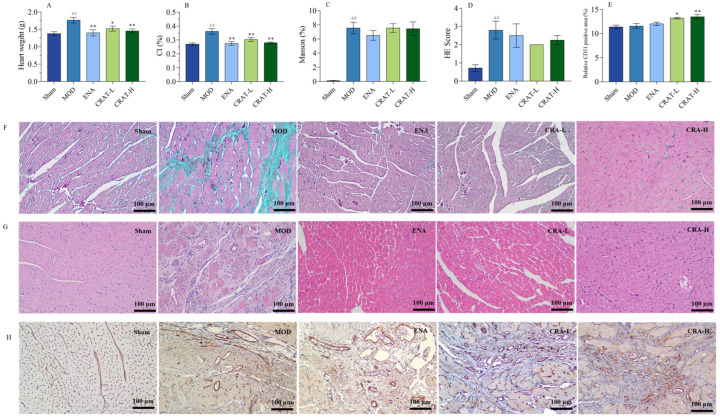
CRAT can improve the organic damage of heart failure caused by coronary artery ligation. (**A**) Heart weight, (**B**) CI. The left ventricle sections were studied with Masson’s trichrome staining, (**C**) Mason staining, (**D**) H&E Score, (**E**) Relative CD31 positive area, (**F**) H&E staining, and Scale bars, 100 μm. (**G**) Immunohistochemistry of CD31, and with corresponding quantification data (**H**). ## *p* < 0.01 vs. Sham; * *p* < 0.05, ** *p* < 0.01 vs. MOD. *n* = 8 per group.

Chronic heart failure leads to physiological and pathological changes such as cardiac hypertrophy, ventricular wall thickening, and ventricular remodeling. After coronary ligation, the model control group of rats (Figure 8A,B) exhibited a significant increase in heart weight and cardiac index. After 5 weeks of treatment, all treatment groups showed a significant reduction in both heart weight and cardiac index. These results indicate that CRAT in various dosages can significantly improve myocardial hypertrophy in heart failure, with a dose–response relationship.

#### 2.3.4. CRAT Can Improve Myocardial Fibrosis and Inflammation in Heart Failure Rats with Retained Chronic Heart Failure Induced by Coronary Artery Ligation

With 5 weeks of consecutive treatment, the ENA group and all CRAT treatment groups showed a decreasing trend in collagen fiber production. The study results also showed that the model control group rats had a significant increase in H&E staining scores, indicating extensive left ventricular myocardial injury, cartilaginous and bony metaplasia, mineralization, left ventricular dilation, ventricular wall atrophy, thinning, and a large area of inflammatory cell infiltration. The ENA group and all CRAT treatment groups were able to reduce the area of left ventricular injury, improve conditions of cartilaginous and bony metaplasia and mineralization, and decrease the area of inflammatory cell infiltration (Figure 8). These results indicate that the various treatment groups can protect myocardial cells and ameliorate the degree of myocardial fibrosis and inflammation.

#### 2.3.5. CRAT Can Increase Myocardial Neovascularization

CD31 is commonly expressed in vascular endothelial cells and plays a critical role in angiogenesis. According to the results of CD31 staining, both CRAT-L and CRAT-H groups showed a significant increase in the percentage of CD31 positive staining (Figure 8E,H), suggesting that CRAT promotes angiogenesis in rats. This increase in new blood vessel formation enhances myocardial blood supply and oxygenation, thereby ameliorating the conditions of chronic heart failure rats.

## 3. Discussion

Gardenia is a medicinal herb known for its dual purposes in both culinary and medicinal contexts, with its extract, crocetin, demonstrating broad pharmacological actions and good safety. While extensive studies have been carried out on the pharmacological effects of crocetin, there is relatively less research on its application in treating heart failure. In the United States, clinical studies on crocetin for stroke treatment indicate its potential importance in mitigating ischemic hypoxia. Chronic heart failure, characterized by impaired cardiac contractility and reduced ejection, leads to insufficient systemic blood supply, causing symptoms like dizziness, fatigue, and breathing difficulties, severely threatening life. The prevalence of heart failure increases with age in developed countries [48,49]. Globally, there are about 63 million people with heart failure, with HFpEF patients constituting about 50% [50]. The incidence of HFpEF in China is 3.5%, characterized by high morbidity and mortality rates [51]. The 2020 China Heart Failure Medical Quality Control Report analyzed 33,413 records of in-hospital heart failure patients from 113 hospitals across the country from January 2017 to October 2020, revealing a 2.8% [52,53] mortality rate among hospitalized patients.

Crocetin exhibits various pharmacological actions [54,55,56], including hypoglycemic and lipid-lowering effects, as well as anti-myocardial ischemia, antioxidant stress, and anti-apoptosis effects, and this study found that crocetin can protect the heart and improve chronic heart failure by regulating multiple pathological processes. EF, FS, and SV are direct indicators of cardiac contractile function. EF reflects cardiac contractile function and ejection capacity, while FS reflects the heart’s radial contractile ability [57]. In models of chronic heart failure induced by abdominal aorta constriction, renal hypertension, and coronary ligation, all groups showed a significant decrease in EF and FS, consistent with literature reports [58,59], indicating that increased cardiac afterload and myocardial ischemia severely affect normal cardiac ejection function, a common cause of chronic heart failure. The study results suggest that crocetin can significantly increase EF, FS, and SV, thereby improving chronic heart failure. However, with the progression of heart failure, short-term administration of crocetin is insufficient to effectively improve cardiac ejection capacity. The results imply that treatment with crocetin for chronic heart failure is a long-term process, while short-term treatment can delay the onset of heart failure but is not ideal for treating the condition.

This study also explored two models of chronic heart failure induced by abdominal aorta constriction and renal hypertension, using both therapeutic and preventative treatment plans. The results show that preventative treatment can significantly delay the progression of heart failure, indicating that earlier intervention can lead to better therapeutic outcomes. LVDd and LVDs are indirect indicators of cardiac ejection function, and their increase suggests left ventricular dilation, indicative of congestive heart disease. In both load-induced and ischemic-induced heart failure models, an increase in left ventricular diameter is observed, along with myocardial cell hypertrophy, inflammatory cell infiltration, necrosis, and myocardial fibrosis, a key pathological feature of HFpEF [60,61,62] and a major cause of cardiac hypertrophy, myocardial stiffness, and ventricular diastolic dysfunction, affecting the prognosis of HFpEF. Therefore, inhibiting myocardial fibrosis is important for improving HFpEF and prognosis. The results show that administration of crocetin significantly reduces the degree of myocardial fibrosis, decreases inflammatory cells, and inhibits myocardial cell hypertrophy, suggesting that crocetin can improve heart failure by anti-fibrotic effects and inhibiting myocardial remodeling.

Animal models of heart failure have been invaluable in advancing our understanding of the pathophysiology of the disease, as well as in the development and testing of potential therapeutics. However, it is important to recognize the limitations of these models in order to accurately extrapolate their findings to the clinical setting. Some of the key limitations include [63,64]: (i) Genetic and physiological differences: Animals and humans have distinct genetic backgrounds and physiological responses to diseases. As a result, the response of animal models to heart failure may not perfectly reflect the clinical presentation of the disease in humans. (ii) Comparative organ size and function: The size and function of the heart can vary significantly between species, which may affect the translation of findings from animal models to humans. For example, the relative importance of neurohormonal pathways may differ, influencing the efficacy of interventions that target these pathways. (iii) Lifespan and disease progression: The lifespan of animals is typically shorter than that of humans, which may not allow for the full development of the clinical manifestations of heart failure. Additionally, the rate of disease progression may differ between animals and humans, potentially affecting the timing and efficacy of interventions. (iv) Environmental factors: Animals may be exposed to a different set of environmental factors than humans, which could influence the development and progression of heart failure. Despite these limitations, animal models of heart failure continue to provide valuable insights that can be applied to the clinical setting. To enhance the translation of findings from animal studies to humans, researchers often employ a multi-modal approach that includes human clinical trials, genetic studies, and computational modeling. By combining data from multiple sources, researchers can gain a more comprehensive understanding of heart failure pathophysiology and develop more effective diagnostic and therapeutic strategies.

In chronic heart failure, myocardial injury, ventricular remodeling, and alterations in mitochondrial structure and number occur, leading to a shift in myocardial metabolism from fatty acids to glucose, decreased glucose oxidation, and increased glycolysis [46]. TG hydrolysis, one of the pathways for free fatty acid production, decreases after heart failure, leading to increased serum levels [46]. The study suggests that crocetin can regulate lipid and energy metabolism to some extent and promote angiogenesis, increasing oxygen supply and further powering myocardial contraction. Thus, crocetin may improve chronic heart failure by regulating lipid and energy metabolism and increasing vascular oxygen supply.

## 4. Material and Methods

### 4.1. Drug and Reagents

Crocetin (CRA) and its sodium salt, Disodium trans-crocetinate (CRAT), were supplied by Boji Medical & Pharmaceutical Technology Co., Ltd. (Guangzhou, China). Enalapril Maleate tablets (Lot. 22012712) were acquired from Guangdong Bidi Pharma Co., Ltd. (Guangzhou, China). Sodium Carboxymethyl Cellulose (CMC-Na, CAT# 30036328) was purchased from Sinopharm Chemical Reagent Co., Ltd. (Shanghai, China). Next, 0.9% Sodium Chloride Injection (Lot. 20082233) was obtained from Xinhai Yuan Sheng Pharmaceutical Co., Ltd. (Shanghai, China). Isoflurane (CAT# R510-22-10) was sourced from Shenzhen RWD Life Science Co., Ltd. (Shenzhen, China). Injectable Penicillin Sodium (Lot. 20220317) was procured from North China Pharmaceutical Group Corporation Animal Health Products Co., Ltd. Sutent (Lot. 8G4VA) was purchased from Pfizer (Paris, France). Dormicum (Lot. 20210401) was acquired from Dunhua Sainty. NT-ProBNP assay kits (CAT# CSB-E08752r) were obtained from Wuhan Huamei Biotech Co., Ltd. (Wuhan, China). Catalase (CAT, CAT# A007-2-1) assay kits and Glucose assay kits (CAT# F006-1-1) were purchased from the Nanjing Jiancheng Bioengineering Institute (Nanjing, China).

### 4.2. Instruments

The research utilized an array of instruments including a Sartorius electronic balance (BP211D), a Thermo benchtop centrifuge (ST16R), a Biopac 16-channel physiological recorder (MP150), a VentElite respirator (55-7040), a Chengdu Taimeng bi-signal collection and analysis system (BL420F), a Xinghua Tongchang constant temperature animal surgery table (DWV-Ⅱ-HW), a BioTek microplate reader (ELX808), a Nanjing Supenda anesthetic respirator (DM6B), a Baisheng Medical color Doppler ultrasound device (Sigma Vet), an Esaote color Doppler ultrasound system (MyLab40HD), and a Beijing Softalon Biotechnology non-invasive blood pressure monitor (BP-2010A).

### 4.3. Animals

All animal experiments were approved by the IACUC of the Drug Evaluation Center of Boji Medical Technology Co., Ltd. (AAALAC ACCREDITATION No. 001956). (Guangzhou, China). The study involved Sprague–Dawley (SD) rats, male, aged 6 to 7 weeks, weighing between 190 and 240 g, sourced from Hunan Slack Jingda Laboratory Animal Co., Ltd. (Changsha, China).The animals were housed under controlled environmental conditions: temperature 20–26 °C, humidity 40–70%, a ventilation frequency of at least 15 changes per hour, and a housing density not exceeding 5 rats per cage. The light–dark cycle was maintained at 12 h each. 

### 4.4. Study Design

#### 4.4.1. Preserved Chronic Heart Failure Induced by Coarctation of the Abdominal Aorta in Rats

After a week of acclimatization, the Sprague–Dawley rats were randomly divided into a sham-operated group (Sham) and a model preparation group. Anesthesia was administered via intramuscular injection of a mixture of Sutent and Dormicum (10:1 ratio). The rats were then positioned dorsally and secured on the operating table, with the abdominal area prepared and disinfected for surgery. An incision of about 3 cm was made along the abdominal midline, 2–3 cm below the xiphoid process. Using curved forceps, the abdominal aorta was gently and bluntly dissected and ligated. Postoperative wound disinfection followed, with an intramuscular injection of 50,000 U of penicillin administered immediately after surgery and continued for three consecutive days under routine housing conditions. The sham-operated group underwent the same procedure without the constriction of the abdominal aorta.

**Figure 9:**
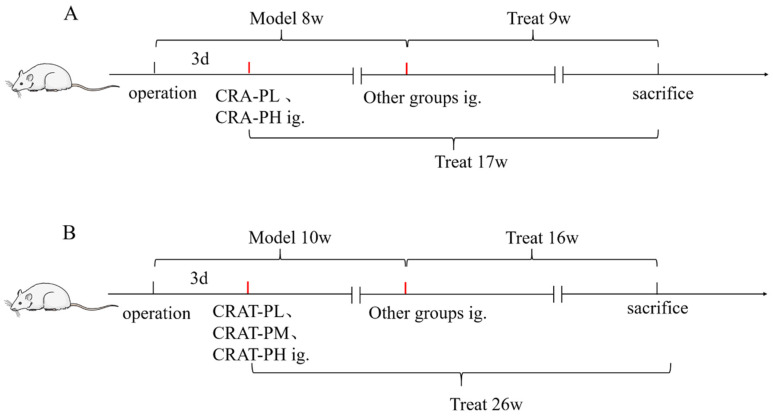
The flow chart of Crocetin administration. (**A**) Flow chart of administration in a chronic heart failure model induced by abdominal aortic coarctation. (**B**) Flow chart of drug administration in chronic heart failure model induced by renal hypertension.

After surgery, the model preparation group was further subdivided into model control (MOD), positive control (Enalapril, ENA), low-dose CRA (CRA-L), high-dose CRA (CRA-H), preventative low-dose CRA (CRA-PL), and preventative high-dose CRA (CRA-PH) groups. Dosage administration included oral gavage of CRA-L and CRA-H at 10 mg/kg and 30 mg/kg, respectively, CRA-PL and CRA-PH at the same respective doses, and the ENA group at 10 mg/kg. The Sham and MOD groups received an equivalent volume of 0.5% Sodium Carboxymethyl Cellulose solution at 10 mL/kg once daily. The preventative groups began treatment on the third day post-modeling, while other groups started 8 weeks post-modeling, continuing for a total of 9 weeks of administration. The preventative groups received 17 weeks of continuous treatment (Figure 9A).

At 8 weeks post-modeling (8 weeks into treatment for the preventative groups), 4 weeks, and 9 weeks into drug administration, cardiac function was assessed. On the final day of the experiment, rat blood samples were collected to measure NT-proBNP levels, and the heart was extracted for Cardiac Index (CI) measurement, as well as H&E and Masson staining.

#### 4.4.2. Preserved Chronic Heart Failure Induced by Renal Hypertension in Rats

Rats were randomly allocated into a sham-operated group (Sham) and a model preparation group. Before modeling, the rats were fasted for at least 12 h with water ad libitum. Anesthesia was induced with a Sutent-Dormicum (10:1) mixture, at a volume of 0.05 mL/250 g. For the model preparation group, the rats were positioned left-side down on the operating table, shaved, and disinfected in the abdominal area. An incision was made 1.5 cm below the xiphoid process along the abdominal midline. At the left renal hilum, the renal artery was isolated, and a sterile suture was passed underneath it. A 0.25 mm diameter acupuncture needle was placed parallel and adjacent to the long axis of the renal artery, and both were ligated with the sterile suture before the needle was withdrawn, creating unilateral renal artery stenosis. The rats were fasted for 24 h post-surgery, followed by ad libitum feeding and water.

The model preparation group was further subdivided into model control (MOD), positive control (Enalapril, ENA), CRAT low dose (CRAT-L), medium dose (CRAT-M), high dose (CRAT-H), preventative low dose (CRAT-PL), and preventative high dose (CRAT-PH) groups, with 10 rats each. The preventative groups began treatment 3 days post-modeling with CRAT-PL and CRAT-PH, respectively, administered 10 mg/kg and 30 mg/kg, calculated as CRA. The treatment duration was consistent across all groups. The remaining groups began treatment 10 weeks post-modeling, with the low-, medium-, and high-dose groups receiving 10, 20, and 40 mg/kg, respectively, and the ENA group receiving 30 mg/kg. The Sham and MOD groups received an equivalent volume of 0.5% CMC-Na at 10 mL/kg once daily. Continuous treatment lasted 16 weeks for the treatment groups and 26 weeks for the preventative groups (Figure 9B).

The blood pressure of all animals was measured under awake conditions before modeling, at 4, 8, and 10 weeks post-modeling, and then every 2, 4, 6, 8, 12, and 16 weeks after drug administration using a non-invasive blood pressure monitor. Cardiac function was assessed at 2, 4, 6, 8, 12, and 16 weeks post-treatment using a color Doppler ultrasound device. Blood samples were collected at 8, 14, and 16 weeks post-treatment to measure NT-proBNP, Total Cholesterol (TC), Triglycerides (TG), Angiotensin II (AngⅡ), and Renin levels. After the conclusion of treatment, the animals were anesthetized, and their hearts were extracted for H&E staining and measurements of Cardiac Index and Left Ventricular Mass Index (LVMI).

#### 4.4.3. Preserved Chronic Heart Failure Induced by Coronary Artery Ligation in Rats

Before surgery (1–2 h), the rats were injected intramuscularly with 0.1 mL of lidocaine per rat to prevent ventricular fibrillation. Anesthesia was then induced using a muscle injection of a Sutent-Dormicum (10:1) mixture, at a volume of 0.05 mL/250 g. The skin was prepared and disinfected with iodophor, and the rats were placed in a supine position. Following tracheal intubation and connection to an animal ventilator, a two-lead electrocardiogram was hooked up to monitor heart activity. A blunt dissection was performed between the 4th and 5th intercostal spaces, where cardiac pulsation was most pronounced, and the pleura was opened to expose the heart. A 6-0 nylon suture with a needle was inserted 1–2 mm below the left atrial appendage and quickly tied around the left anterior descending artery to induce ischemia. The skin was sutured, and 1 mg/kg of meloxicam was administered subcutaneously for analgesia, followed by an intraperitoneal injection of 80,000 units of penicillin sodium for infection prevention. The sham group underwent the same procedures except for the ligation of the coronary artery.

After modeling, the animals were routinely fed until week 8. Rats with an ejection fraction (EF) < 65% were selected for the experiment and randomly divided into five groups: model control (MOD), positive drug control (Enalapril, ENA), low-dose CRAT (CRAT-L), high-dose CRAT (CRAT-H), and a control group (CON) given an equivalent volume of 0.5% CMC-Na. CRAT-L and CRAT-H were administered at 10 and 30 mg/kg (calculated as CRA content), and the ENA group was given 2 mg/kg. Each group received the respective treatment or solvent orally at a volume of 10 mL/kg once daily for five consecutive weeks.

Cardiac function was assessed at weeks 3 and 5 post-treatment, and on the final day of the experiment, blood samples were collected to determine levels of NT-proBNP, glucose (GLU), TC, and TG. Hearts were taken for H&E, Masson staining, and CD31 immunohistochemical staining, as well as for measurements of phosphocreatine (PCr) and catalase (CAT) in cardiac tissue.

### 4.5. Detection by Echocardiography

The rats were anesthetized with isoflurane and placed in a supine position for skin preparation. An 18 MHz probe at a depth of 3 cm was used with a color Doppler ultrasound to measure left ventricular end-diastolic dimension (LVDd), left ventricular end-systolic dimension (LVDs), ejection fraction (EF), fractional shortening (FS), and stroke volume (SV).

### 4.6. Blood Pressure

The measurements were performed by the blood pressure monitor’s operational manual, and the rats’ tail blood flow pulsations were measured three times consecutively to obtain an average value. Blood pressure was measured three days in a row before the experiment, once daily.

### 4.7. Measurement of Cardiac Hypertrophy

After weighing, the rats’ hearts were extracted, washed with cold saline, blotted dry with filter paper, and weighed to determine heart weight (HW). The Cardiac Index (CI) was calculated as HW/BW, and the left ventricular mass (LVM) was determined after removing the right ventricle and atrial tissues, calculating the Left Ventricular Mass Index (LVMI) as LVM/BW. In the abdominal aorta constriction model, the tibia length was measured to assess cardiac hypertrophy by comparing heart weight to tibia length.

### 4.8. H&E

Left ventricular wall tissue was fixed in 4% paraformaldehyde, embedded in paraffin, and sectioned. The sections were then immersed in hematoxylin for 10 min, rinsed under running water, differentiated in 1% hydrochloric acid in ethanol for 2 s, blued in running water for 10 min, and counterstained in eosin for 80 s. They were then washed again, dehydrated in 95% alcohol followed by absolute ethanol, air-dried, and mounted with a cover slip using a mounting medium. The heart tissue was evaluated using H&E staining to score myocardial injury on a semi-quantitative scale (Table 1).

### 4.9. Masson’s Triple Stain

Left ventricular wall tissue sections were deparaffinized to water, stained with Weigert’s iron hematoxylin for 5 min, rinsed under running water, differentiated in 1% hydrochloric acid in ethanol for 2 s, and again rinsed. The sections were then stained with Picro-Sirius Red for 5 min, washed, treated with phosphomolybdic acid solution for 5 min, and counterstained with aniline blue for 3 min. They were rinsed with 1% acetic acid until no more blue dye was released, dehydrated through 95% and absolute ethanol, cleared in xylene, and mounted with a neutral resin. In the abdominal aorta constriction model, Image-Pro Plus software 6.0 was used to calculate the myocardial collagen area, and the collagen volume fraction (CVF) was expressed as the myocardial collagen area/total field area. In the coronary ligation model, IPP 6.0 software analyzed the areas of positive staining and the total areas to calculate the percentage of positive staining: positive staining percentage (%) = area of positive staining/total area × 100.

### 4.10. Immunohistochemical Stain

In the coronary ligation model experiment, left ventricular myocardial tissue was subjected to CD31 immunohistochemical staining. Three images were captured from the infarcted area, the border zone between infarcted and normal tissue, and the normal area, using 200× magnification. The images were analyzed with IPP 6.0 image analysis software to determine the areas of positive staining and total areas. The percentage of positive staining was calculated using the formula: positive staining percentage (%) = area of positive staining/total area × 100. The percentages from the three images were summed to yield the final score for each animal.

### 4.11. Quantification and Statistical Analysis

Statistical analyses were evaluated using SPSS software 22, with results expressed as the mean ± standard deviation (X ± SD). For comparisons of means across multiple groups, a one-way analysis of variance (One-Way ANOVA) was employed. For pairwise comparisons between group means, the Least Significant Difference (LSD) method was used when variances were equal, and Dunnett’s T3 method was applied in cases of unequal variances.

## 5. Conclusions

**Figure 10:**
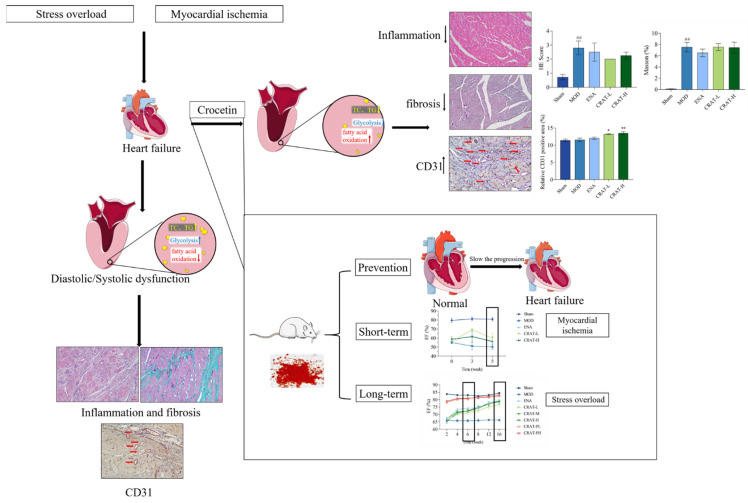
Crocetin can improve chronic heart failure. Schematically illustrated that preventive administration of crocetin can delay the progression of heart failure and long-term administration is more effective than short-term (Red arrows refer to blood vessels, ## *p* < 0.01 vs. Sham; * *p* < 0.05, ** *p* < 0.01 vs. MOD).

In the presented report, we successfully established and evaluated models of load-induced heart failure and myocardial ischemia-induced chronic heart failure through coronary ligation (Figure 10). The results indicate that crocetin is suitable for long-term preventative administration in managing chronic heart failure. In heart failure models induced by increased pre- and after-load and myocardial ischemia, crocetin may improve heart failure by inhibiting myocardial hypertrophy, ameliorating myocardial fibrosis, enhancing myocardial contractility, and consequently increasing ejection volume to sustain normal life activities. Additionally, crocetin may regulate energy and lipid metabolism and promote cardiac angiogenesis, increasing myocardial oxygen supply, and providing energy for cardiac contraction, thereby significantly improving chronic heart failure. The present study demonstrated that crocin can improve the symptoms and function of heart failure rats, but the clinical application of crocin needs further investigation.

## Figures and Tables

**Table 1:** Scoring criteria for myocardial loss.

The Extent of Lesion Damage	Score
0–25%	1
26–50%	2
51–75%	3
76–100%	4

## Data Availability

The study data were stored in the archives of the Boji Pharmaceutical Research Center, Boji Medical Biotechnological Co., Ltd. Guangzhou, China.
